# Hepatic vein Doppler in critically ill patients: a reflection of central venous pressure or right ventricular systolic function?

**DOI:** 10.1186/s12871-022-01872-6

**Published:** 2022-10-29

**Authors:** Hongmin Zhang, Ye Liu, Qing Zhang, Xiaoting Wang, Dawei Liu, Critical Care Ultrasound Study Group (CCUSG)

**Affiliations:** 1 Department of Critical Care Medicine, Peking Union Medical College Hospital, Chinese Academy of Medical Sciences and Peking Union Medical College, 1# Shuai Fu Yuan, Dong Cheng District, Beijing, 100730 China; 2 Department of Medicine, University of Alabama at Birmingham Heersink School of Medicine, Birmingham, AL USA

**Keywords:** Echocardiography, Hepatic vein, Systolic filling fraction, Inferior vena cava, Critically ill

## Abstract

**Background:**

To explore whether hepatic vein systolic filling fraction (SFF) is associated with central venous pressure (CVP) and right ventricular (RV) systolic function in critically ill patients.

**Methods:**

Adult patients admitted to ICU with echocardiographic examination were retrospectively enrolled. Echocardiographic parameters including hepatic vein systolic velocity (S) and diastolic phase velocity (D) and haemodynamic information at the time of echo examination were collected. RV systolic dysfunction was defined as tricuspid annular plane systolic excursion (TAPSE) < 16 mm. SFF was calculated as S/(S + D).

**Results:**

Two hundred four patients were enrolled in this study among whom 40 patients had a CVP ≤5 mmHg, 110 patients had a CVP 6–9 mmHg and 54 patients had a CVP ≥10 mmHg. The three groups had similar S velocity, D velocity and SFF. No correlation between SFF and CVP was found (*r* = − 0.046, *p* = 0.500), but correlation between SFF and TAPSE was noticed (*r* = 0.468, *p* < 0.001). The ROC analysis showed that the area under curve (AUC) of SFF for determining CVP ≥10 mmHg was 0.513 (95% CI: 0.420–0.606, *p* = 0.775), but the AUC of SFF for determining RV systolic dysfunction was 0.759 (95% CI: 0.686–0.833, *p* < 0.001).

**Conclusion:**

Hepatic vein systolic filling fraction is associated with RV systolic function in critically ill patients and is not associated with CVP.

## Background

Venous return and right ventricular (RV) function play an important role in the haemodynamic stability in critically ill patients [1, 2]. They can affect organ perfusion by providing enough volume to left ventricle as well as by various levels of venous pressure [3]. Central venous pressure (CVP), as a readily available indicator of the interaction of the RV function and venous return, has been proved to be associated with organ dysfunction [4, 5]. In recent articles, CVP was used as one of the indicators of venous congestion [6].

Point-of-care ultrasound enables intensivists to visualize the venous anatomy and evaluate blood velocity using Doppler imaging. Hepatic vein (HV) Doppler are among the ultrasound parameters that has the potential to detect venous congestion according to previous studies [3, 7]. Some researchers contended that hepatic vein S wave is greater than D wave with normal CVP, and S wave decreases with an increase of CVP [7]. Nagueh SF et al. concluded that hepatic vein systolic filling fraction (SFF), a ratio of S velocity divided by the sum of S and D velocity, is well correlated with CVP and can be used to estimate CVP [8]. However, there has been inconsistent conclusions about the relationship of hepatic vein Doppler and central venous pressure [9]. Furthermore, RV motion determines the shape of hepatic vein S wave and D wave. During ventricular systole the tricuspid annulus moves toward the cardiac apex, which creates a relative negative pressure in the atrium and causes antegrade blood flow from the hepatic vein into the heart [10]. We thus hypothesize that SFF is a reflection of RV systolic function rather than CVP. To date, few studies have examined the association between hepatic vein Doppler and RV systolic function. Therefore, the goals of this study are to demonstrate whether SFF is associated with CVP and RV systolic function in critically ill patients.

## Patients and methods

### Study population

We retrospectively studied a cohort of adult patients admitted to intensive care unit (ICU) from 1 May 2019 to 1 March 2022. Patients who had undergone echocardiographic examination due to shock differentiation, volume responsiveness assessment, heart function appraisal, or hypoxia differentiation were included.

Patients with the following conditions were excluded from the study: lack of echo images on inferior vena cava (IVC) or HV Doppler; lack of CVP measurement; severe tricuspid regurgitation or stenosis; tricuspid replacement or tricuspid annuoplasty; non-sinus rhythm.

The study was conducted in compliance with the Declaration of Helsinki and was approved by the ethics committee of Peking Union Medical College Hospital, Beijing, China (Approval No. ZS-1602). Informed consent was waived given its retrospective nature.

### Echocardiography

Echocardiograms were recorded within the first 24 hours of ICU admission using an echocardiograph by two intensivists who were experienced in echocardiography. Electrocardiograms were recorded continuously during the echocardiographic examination. Images were saved for offline analysis.

**Fig. 1:**
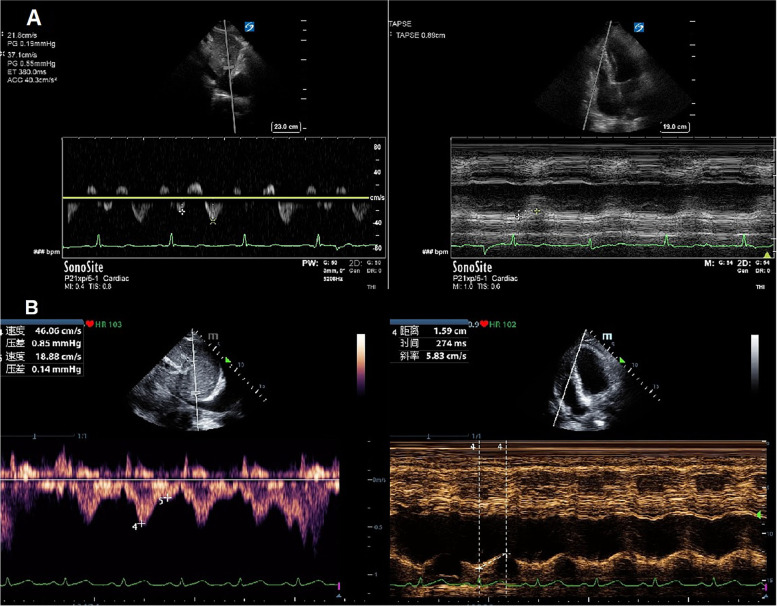
The measurement of SFF and TAPSE in two patients both with CVP 8 mmHg. **A** A patient whose S wave was 21.8 cm/s, D wave was 37.1 cm/s, and TAPSE was 8.9 mm; **B** A patient whose S wave was 40.1 cm/s, D wave was 18.9 cm/s, and TAPSE was 15.9 mm. SFF: hepatic vein systolic filling fraction; TAPSE: tricuspid annular plane systolic excursion; CVP: central venous pressure

The left ventricular ejection fraction (LVEF) was obtained using a modified biplane Simpson’s method from apical two- and four-chamber views. TAPSE was also obtained from the apical 4-chamber view by positioning the M-mode cursor along the lateral part of the tricuspid valve ring [11]. The LVOT velocity-time integral (VTI) was obtained from pulsed Doppler imaging by positioning the sample volume at the LVOT approximately 0.5 cm below the aortic valve [12]. Stroke volume (SV) was calculated as π × (LVOT diameter/2)^2^ × VTI. The diameter of the inferior vena cava (IVCD) was measured in the subcostal longitudinal plane, just upstream of the origin of the suprahepatic vein. The middle HV was identified from mid-subcostal view as prior studies described [7, 10]. To obtain HV PW Doppler, a phased array transducer was used with a sample volume of 2-3 mm and a velocity range of 80-100 mm/s. It was interrogated 2-3 cm from its junction to the IVC. Color flow Doppler was used to identify high flow parallel to the ultrasound beam and then pulsed wave Doppler was obtained at the end respiratory phase (Fig. 1A, B). Hepatic vein SFF was calculated as peak systolic velocity (S) divided by the sum of peak systolic velocity and peak diastolic velocity (D) [8]. Although the SFF as a ratio can mitigate the problem to find the adequate angle, we managed to align the HV blood flow with the ultrasound beam. The ratio of RV end-diastolic area and LV end-diastolic area (R/LVEDA) was obtained at the end of ventricular diastole. Right ventricular systolic dysfunction (RVSD) was defined as TAPSE < 16 mm [13, 14].

### Other parameters collected

Demographic information, Acute Physiology and Chronic Health Evaluation (APACHE) II scores, Sequential Organ Failure Assessment (SOFA) scores, diagnosis of shock or respiratory failure and comorbidities. We also recorded each patient’s heart rate (HR), mean arterial pressure (MAP) ventilator settings, and need of vasopressor infusion at the time of the echocardiographic examination. We also collected prognostic data including creatinine, need of continuous veno-venous hemofiltration, length of ICU stay and ICU mortality.

### Statistical analysis

We performed the statistical analysis using the statistical software package SPSS 22.0 (IBM Corp. Released 2013. IBM SPSS Statistics for Windows, Version 22.0. Armonk, NY: IBM Corp). Continuous data were expressed as the mean ± SD or the median and the interquartile range. Categorical variables were presented as frequency and percentages. The distributions of the continuous values were assessed for normality by the Kolmogorov-Smirnov test. Differences between groups were compared by Student’s t-test, the Mann-Whitney U test, the chi-squared test, or Fisher’s exact test where appropriate. Pearson’s correlation coefficients and their corresponding *p* values were calculated to assess the variable relationships. Receiver-operating characteristic (ROC) curves were generated and the area under curve (AUC) were then calculated. Intraobserver and interobserver variability in TAPSE, SFF and IVCD were assessed in 20 randomly selected patients and were tested using intraclass correlation coefficients (ICCs). An ICC > 0.8 was considered excellent agreement. All *p*-values were two tailed and were considered significant for *p* < 0.05.

## Results

### Baseline characteristics of the study population

A total of 735 patients who had echocardiographic examination were screened for enrolment. Two hundred seventy patients were excluded due to the lack of IVCD or HV Doppler measurement; 197 were excluded due to lack of CVP monitoring; 18 were excluded due to severe TR; 10 were excluded due to TV replacement or TV plasty; 36 were excluded due to non-sinus rhythm. Two hundred four patients were enrolled in this study among whom 40 patients had a CVP ≤5 mmHg, 110 patients had a CVP 6–9 mmHg and 54 patients had a CVP ≥10 mmHg. The three groups had similar age, sex proportion, APACHE II, comorbidities, proportion of respiratory failure, fluid administration, serum creatinine, proportion of CVVH and length of ICU stay. Patients with CVP ≤5 mmHg had lower SOFA, lower proportion of shock and vasopressor use, lower proportion of MV support and lower ICU mortality than patients with CVP ≥10 mmHg (*p*<0.05)(Table 1).

**Table 1:** Baseline characteristics of the study population

Categories	All patients (*n* = 204)	CVP ≤ 5 (*n* = 40)	CVP 6–9 (*n* = 110)	CVP ≥ 10 (*n* = 54)	*p*
Age (yr)	62 (50, 71)	61 (52, 69)	63 (50, 71)	62 (50, 72)	0.968
Sex (male, %)	125 (61.3%)	24 (40.0%)	71 (64.5%)	30 (55.6%)	0.531
APACHEII	21 (16, 27)	21 (17, 26)	20 (15, 25)	21 (17, 26)	0.208
SOFA	11 (9, 14)	10 (8, 12)	11 (9, 13)	13 (10, 16)	0.019^b^
Diagnosis (n, %)					
Shock	91 (44.6%)	9 (22.5%)	51 (46.4%)	31 (57.4%)	0.003^b^
Respiratory failure	33 (16.2%)	7 (17.5%)	19 (17.3%)	7 (13.0%)	0.756
Comorbidities (n, %)					
HTN	83 (40.7%)	15 (37.5%)	44 (39.1%)	24 (44.4%)	0.777
DM	68 (23.3%)	13 (32.5%)	39 (35.5%)	16 (29.6%)	0.753
CAD	47 (23.0%)	9 (22.5%)	29 (26.4%)	9 (16.7%)	0.381
CKD	8 (3.9%)	1 (2.5%)	5 (4.5%)	2 (3.7%)	0.846
COPD	9 (4.4%)	1 (2.5%)	7 (6.4%)	1 (1.9%)	0.336
MV (n, %)	159 (77.9%)	18 (45.0%)	92 (83.6%)	49 (90.7%)	< 0.001^b b, C^
^*^Fluid administration (ml)	3430 (2388, 4236)	2938 (2301, 4361)	3430 (2535, 4147) 707)	3561 (2213, 4279)	0.608
Vasopressor (n, %)	136 (66.7%)	19 (47.5%)	72 (65.5%)	45 (83.3%)	0.001^b^
Scr (μmol/L)	93 (67, 152)	94 (65, 150)	87 (68, 137)	94 (64, 178)	0.396
CVVH (n, %)	37 (18.1%)	6 (15.0%)	18 (16.4%)	13 (24.1%)	0.411
ICU stay (day)	7 (4, 12)	7 (5, 15)	6 (4, 15)	6 (3, 10)	0.362
ICU mortality (n, %)	35 (17.2%)	2 (5.0%)	20 (18.2%)	13 (24.1%)	0.048^b^

*APACHE* acute physiology and chronic health evaluation, *SOFA* sequential organ failure assessment, *HR* heart rate, *HTN* hypertension, *DM* diabetes mellitus, *CAD* coronary arterial disease, *CKD* chronic kidney disease, *COPD* chronic obstructive pulmonary disease, *PEEP* positive end-expiratory pressure, *Pplat* plateau pressure, *Scr* serum creatinine, *CVVH* continuous veno-venous hemofiltration, *ICU* intensive care unit

^*^fluid administered within 24 hours before echo examination

^a^CVP ≤ 5 vs. CVP 6–9, *p* < 0.05

^b^CVP ≤ 5 vs. CVP ≥ 10, *p* < 0.05

^c^CVP 6–9 vs. CVP ≥ 10, *p* < 0.05

### Haemodynamic and echocardiographic parameters of the patients

**Fig. 2:**
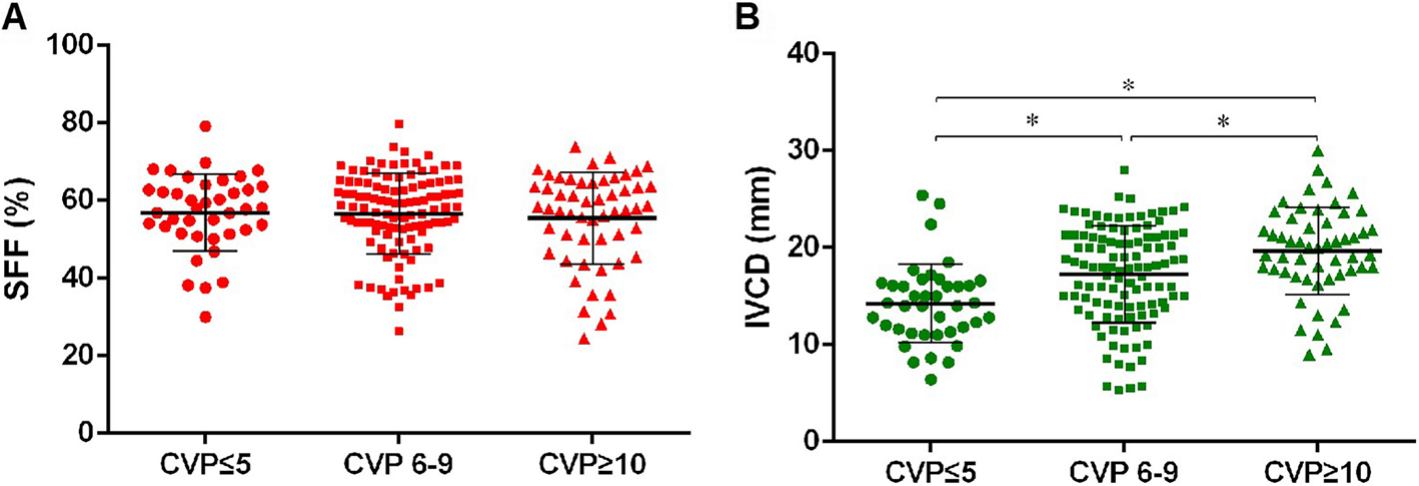
SFF and IVCD in three groups. **A** There was no difference in SFF value among the three groups, *p* = 0.732; **B** The three groups had different IVCD value, *p* < 0.001. *p<0.05. SFF: hepatic vein systolic filling fraction; TAPSE: tricuspid annular plane systolic excursion; CVP: central venous pressure

The three groups had similar HR and MAP. No significant difference was found among the three groups regarding LVEF, TAPSE, FAC, R/LVEDA, E wave velocity, e’ velocity and SVI. The IVCD was significantly different among the three groups (*p* < 0.001). The three groups had similar HV S velocity, HV D velocity and SFF (Table 2, Fig. 2A, B).

**Table 2:** Haemodynamic and echocardiographic parameters

Categories	All patients (*n* = 204)	CVP ≤ 5 (*n* = 40)	CVP 6–9 (*n* = 110)	CVP ≥ 10 (*n* = 54)	*p*
HR (bpm)	93 ± 18	90 ± 16	92 ± 17	97 ± 22	0.109
MAP (mmHg)	81 ± 12	81 ± 13	82 ± 12	79 ± 12	0.386
CVP (mmHg)	8 (6, 10)	4 (3, 5)	8 (7, 8)	12 (10, 13)	< 0.001^a, b, C^
TAPSE (mm)	18.1 ± 5.5	18.2 ± 5.4	17.9 ± 5.3	18.4 ± 6.0	0.843
FAC (%)	47 (35, 55)	47 (41, 56)	47 (36, 54)	47 (32, 53)	0.297
R/LVEDA	0.50 (0.44, 0.61)	0.49 (0.42, 0.60)	0.50 (0.43, 0.60)	0.53 (0.46, 0.66)	0.151
TR (m/s)	2.4 (2.1, 2.6)	2.3 (2.0, 2.5)	2.3 (2.1, 2.6)	2.4 (2.2, 2.6)	0.097
LVEF (%)	56 (45, 66)	56 (40, 62)	56 (45, 66)	59 (44, 67)	0.675
E (cm/s)	61.7 (51.8, 81.9)	57.8 (43.1, 72.4)	63.3 (53.4, 80.2)	61.5 (52.4, 87.1)	0.087
e’(cm/s)	7.5 (5.8, 9.6)	8.1 (5.7, 9.8)	7.0 (5.5, 10.4)	7.8 (6.3, 10.1)	0.410
SVI (ml/m^2^)	35.0 (28.1, 45.4)	36.4 (29.4, 44.4)	33.9 (27.7, 45.4)	35.8 (25.2, 46.2)	0.954
IVCD (mm)	17.7 (13.9, 21.0)	14.2 (11.4, 16.3)	18.0 (13.9, 21.2)	20.0 (17.4, 22.6)	< 0.001^a, b, C^
HV S (cm/s)	27.9 (20.0, 38.7)	32.0 (22.3, 40.8)	26.0 (19.0, 38.9)	26.9 (19.8, 36.5)	0.638
HV D (cm/s)	20.1 (17.0, 26.9)	22.3 (18.0, 28.0)	19.1 (16.3, 25.0)	21.2 (17.0, 27.3)	0.489
S/D	1.4 (1.1, 1.8)	1.4 (1.1, 1.7)	1.4 (1.1, 1.8)	1.4 (1.0, 1.8)	0.844
SFF (%)	58 (51, 64)	58 (52, 63)	58 (52, 65)	58 (49, 65)	0.732

*HR* heart rate, *MAP* mean arterial pressure, *CVP* central venous pressure, *TAPSE* tricuspid annular plane systolic excursion, *R/LVEDA* ratio of right and left end-diastolic area, *FAC* fractional area change, *TR* tricuspid regurgitation, *LVEF* left ventricular ejection fraction, *E* mitral inflow E wave velocity, *e’* mitral e’ velocity, *SVI* stroke volume index, *IVCD* diameter of inferior vena cava, *HV* hepatic vein, *SFF* hepatic vein filling fraction

^a^CVP ≤ 5 vs. CVP 6–9, *p* < 0.05

^b^CVP ≤ 5 vs. CVP ≥ 10, *p* < 0.05

^c^CVP 6–9 vs. CVP ≥ 10, *p* < 0.05

### Correlation analysis of SFF and CVP and RV function

**Fig. 3:**
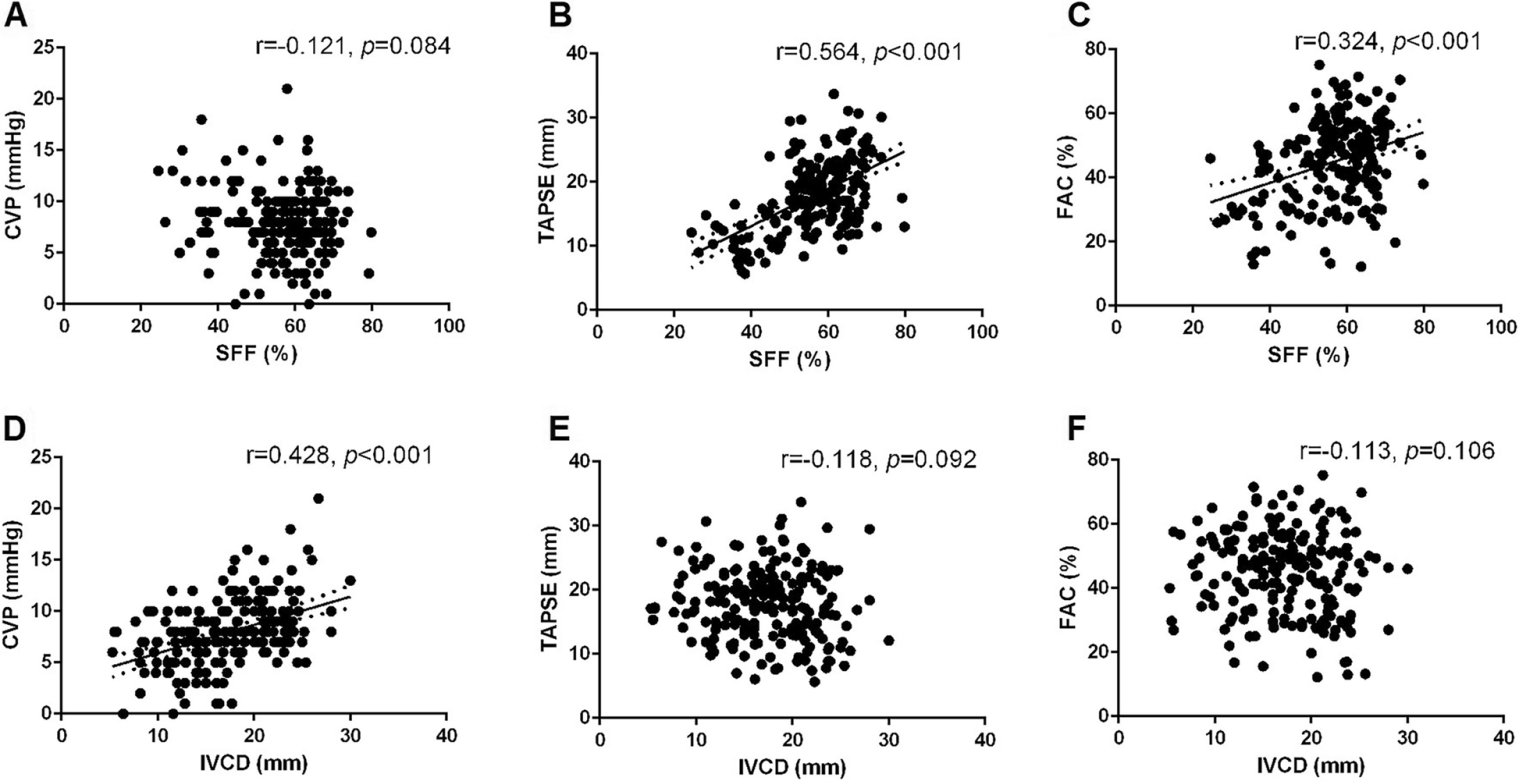
Correlation analysis. **A** Correlation of SFF and CVP in all patients, *r* = − 0.121, *p* = 0.084. **B** Correlation of SFF and TAPSE in all patients, *r* = 0.564, *p* < 0.001. **C** Correlation of SFF and FAC in all patients, *r* = 0.324, *p* < 0.001. **D** Correlation of IVCD and CVP in all patients, *r* = 0.428, *p* < 0.001. **E** Correlation of IVCD and TAPSE in all patients, *r* = − 0.118, *p* = 0.092. **F** Correlation of IVCD and FAC in all patients, *r* = − 0.113, *p* = 0.106. SFF: hepatic vein systolic filling fraction; TAPSE: tricuspid annular plane systolic excursion; CVP: central venous pressure; IVCD: inferior vena cava diameter; FAC: fractional area change

No correlation between SFF and CVP was found in all patients, *r* = − 0.121, *p* = 0.084. SFF was associated with TAPSE (*r* = 0.564, *p* < 0.001) and FAC (*r* = 0.324, *p* < 0.001) (Fig. 3A-C).

### Correlation analysis of IVCD and CVP and RV function

IVCD was associated with CVP, *r* = 0.428, *p* < 0.001. IVCD was not associated with TAPSE (*r* = − 0.118, *p* = 0.092) and FAC (*r* = − 0.113, *p* = 0.106) (Fig. 3D-F).

### ROC analysis of SFF and IVCD for the detection of CVP ≥10 mmHg and RV sytolic dysfunction

To evaluate the sensitivity and specificity of SFF and IVCD for assessing CVP and RV systolic dysfunction, we generated ROC curves. The ROC analysis showed that the AUC of SFF for determining CVP ≥10 mmHg was 0.513 (95% CI: 0.420–0.606, *p* = 0.775); and the AUC of IVCD for determining CVP ≥10 mmHg was 0.685 (95% CI: 0.604–0.766, *p* < 0.001).

**Fig. 4:**
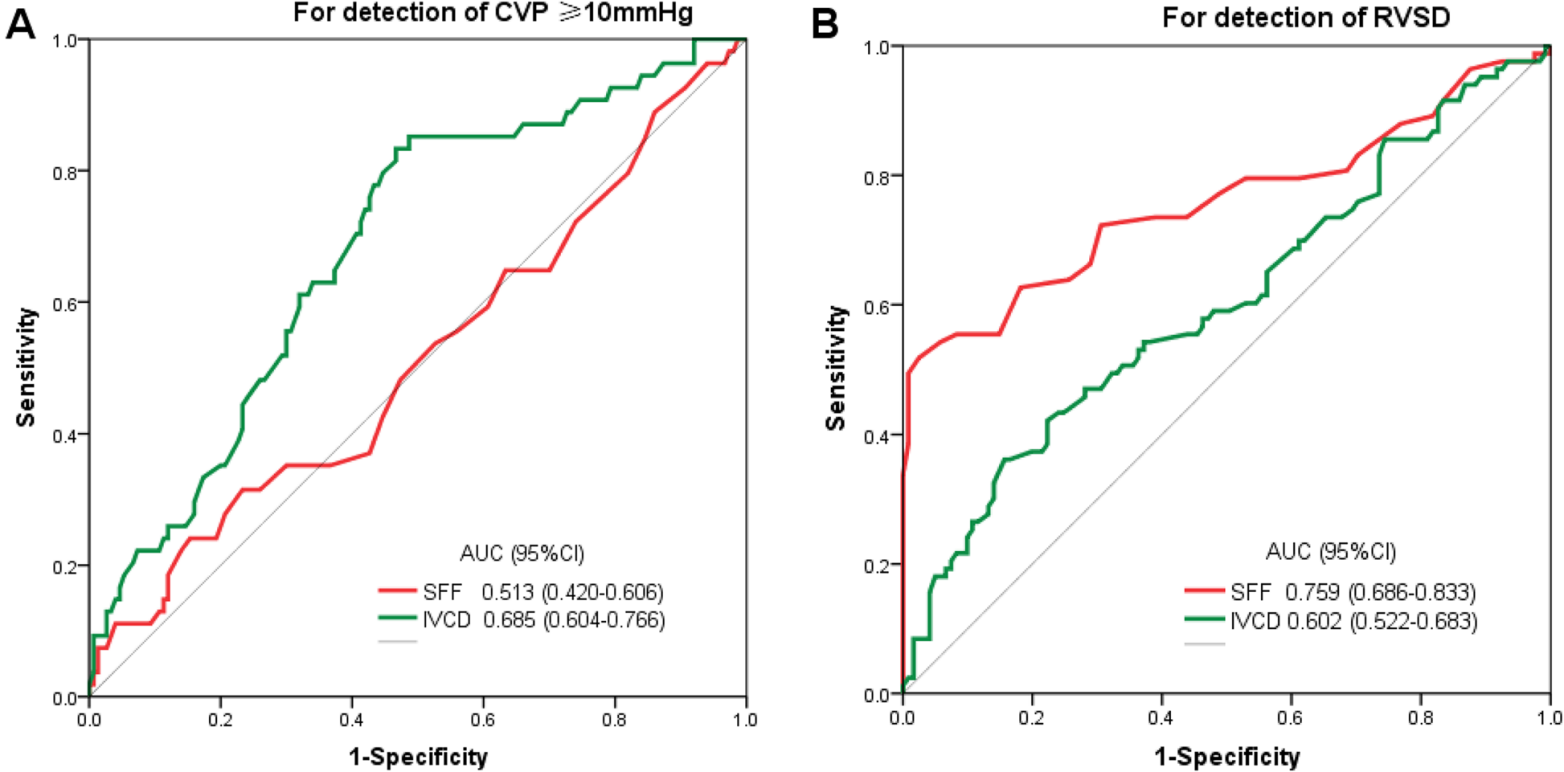
ROC analysis of SFF and IVCD for the detection of CVP over 10 mmHg and RV systolic dysfunction. **A** The area under the curve (AUC) of SFF and IVCD for the detection of CVP>10 mmHg were 0.513 (95% CI: 0.420–0.606; *p* = 0.775) and 0.685 (95% CI: 0.604–0.766; *p* < 0.001). **B** The area under the curve (AUC) of SFF and IVCD for the detection of RVSD were 0.759 (95% CI: 0.686–0.833; *p* < 0.001) and 0.602 (95% CI: 0.522–0.683; *p* = 0.013). SFF: hepatic vein systolic filling fraction; IVCD: diameter of inferior vena cava; CVP: central venous pressure; RV: right ventricle; RVSD: right ventricular systolic dysfunction. AUC: area under the curve

The ROC analysis showed that the AUC of SFF for determining RVSD was 0.759 (95% CI: 0.686–0.833, *p* < 0.001); and the AUC of IVCD for determining RVSD was 0.602 (95% CI: 0.522–0.683, *p* = 0.013) (Table 3, Fig. 4A-B).

**Table 3:** ROC curve analysis

Categories	AUC	95%CI	*p*	Optimum cutoff	Sen	Spe	PPV	NPV
For detection of CVP ≥10								
SFF (%)	0.513	0.420–0.606	0.775	60.6	40.7	60.7	27.2	74.0
IVCD (mm)	0.685	0.604–0.766	< 0.001	18.9	61.1	68.0	40.6	82.9
For detection of RV systolic dysfunction								
SFF (%)	0.759	0.686–0.833	< 0.001	54.5	62.7	81.8	70.2	76.1
IVCD (mm)	0.602	0.522–0.683	0.013	18.7	50.6	66.1	50.6	59.9

*ROC* receiver operating characteristic curve, *AUC* area under curve, *PPV* positive predictive value, *NPV* negative predictive value, *SFF* hepatic vein filling fraction, *IVCD* diameter of inferior vena cava, *CVP* central venous pressure

### Sensitivity analysis

We performed sensitivity analysis by analyzing the correlation of SFF and CVP in patients on MV (*n* = 159) and in patients without MV support (*n* = 45) separately, and found that SFF was still not associated with CVP (*r* = − 0.070, *p* = 0.378 and *r* = − 0.138, *p* = 0.368, respectively).

We also performed sensitivity analysis on the correlation of SFF and TAPSE in patients with normal LVEF and low LVEF. Among patients with LVEF< 50% (*n* = 70), SFF was not associated with LVEF (*r* = 0.158, *p* = 0.191), but was associated with TAPSE (*r* = 0.669, *p* < 0.001); among patients with LVEF ≥50% (*n* = 134), SFF was not associated with LVEF (*r* = 0.156, *p* = 0.073), but was still associated with TAPSE (*r* = 0.414, *p* < 0.001).

### Reproducibility

The intraobserver variabilities in SFF, IVCD, and TAPSE were minimal. The interobserver variability analysis revealed that ICCs for SFF, IVCD, and TAPSE were: 0.905 (95% CI: 0.704–0.965), 0.940 (95% CI: 0.844–0.977) and 0.961 (95% CI: 0.905–0.984), respectively.

## Discussion

In this study, we investigated the relationship among SFF and CVP and RV systolic function. We found that SFF is not correlated with CVP; By contrast, SFF is associated with RV systolic function in these patients. We also found that IVCD was associated with CVP but not with RV systolic function.

Our results suggested that SFF cannot be used to estimate CVP in critically ill patients. This finding is inconsistent with the study by Nagueh SF et al., which concluded that SFF and CVP were well correlated and SFF can be used to estimate CVP in patients with or without MV [8]. The sample volume in the present study was much bigger than theirs. Other researchers also found that SFF and CVP correlated poorly [9]. Pinsky pointed out that RV normally fills below its unstressed volume in which state CVP change might occur without change in RV stretch [15]. In this case, CVP change might be a reflection of intrathoracic pressure or pericardial pressure instead of RV function. In contrast, the HV S wave occurs at systolic phase, so the S velocity is related to RV systolic function to some extent.

IVCD, clearly not perfect, was a more robust parameter in predicting CVP than hepatic vein Doppler. Increased intrathoracic pressure induced by mechanical ventilation or lung hyperinflation can result in IVC dilation [16]. In certain circumstances, CVP could increase without corresponding RV transmural pressure change and RV function alteration. This could be one of the reasons that IVCD was associated with CVP while SFF was not. IVCD correlated with CVP was proved by many researchers [17–19]. SFF has been proposed as a parameter to estimate CVP [13, 20], but this study noticed that it has the potential to alert physicians the existence of RV dysfunction. Higher CVP is usually deemed as an indicator of RV dysfunction [21]. This study revealed that, when CVP is low, decreased SFF might serve as a sign of RV systolic dysfunction.

Veinous congestion has been discussed in the critically ill patients, and hepatic vein Doppler S to D ratio was deemed as an indicator of venous congestion severity [7]. However, this study reminded us that hepatic vein S to D reversal might be more a reflection of RV function rather than CVP or venous congestion, since CVP elevation is a necessity for venous congestion [6]. Vieillard-Baron A et al. reported that RV dilation in combination with CVP increase could be seen as a sign of RV failure and systematic congestion [6]. We found that neither SFF nor IVCD was correlated with R/LVEDA. Thus, further study is still warranted to investigate the relationship of SFF and venous congestion.

## Limitations

This study has several limitations. First, this is a single centre retrospective study, and we only incorporate patients with echocardiographic study and CVP monitoring, thus the enrolment pattern might introduce a selection bias. Second, this study is restricted to patients with sinus rhythm. Thus, the conclusion cannot be applied to patients with arrhythmia. Third, we did not measure IVC variation parameters. IVC variation depends on a few factors including the intrathoracic pressure, the abdominal pressure as well as central venous pressure [22]. Because the intrathoracic pressure change was different in patients on MV and in those without MV, we thus did not incorporate the IVC variation. A previous study demonstrated that IVCD is more robust than IVC collapsibility in the estimation of CVP [23]. Lastly, we excluded patients with severe TR, which could result in reversed S waveform of hepatic vein Doppler. But we only observed a small proportion of patients presenting with severe TR in this study and whether reversed S wave could reflect more severe venous congestion and higher CVP level need to be clarified in future study. Despite these limitations, this study demonstrated that SFF is more a reflection of RV systolic function rather than CVP level in critically ill patients.

## Conclusions

Hepatic vein systolic filling fraction is associated with RV systolic function in critically ill patients and is not associated with CVP. Further study regarding venous congestion is needed.

## Data Availability

All datasets used and/or analysed during the current study are available from the corresponding author on reasonable request.

## Abbreviations

RV: Right ventricle
CVP: Central venous pressure
SFF: Hepatic vein systolic filling pressure
IVCD: Diameter of inferior vena cava
TAPSE: Tricuspid annular plane systolic excursion
LVEF: Left ventricular ejection fraction
VTI: Velocity-time integral
ICU: Intensive care unit
LVOT: Left ventricular outflow tract
R/LVEDA: Ratio of right ventricular end diastolic area and left ventricular end diastolic area
RVSD: Right ventricular systolic dysfunction
APACHE: Acute physiology and chronic health evaluation
SOFA: Sequential organ failure assessment
HR: Heart rate
MAP: Mean arterial pressure
ROC: Receiver-operating characteristic
ICC: Intraclass correlation coefficient
PEEP: Positive end-expiratory pressure
AUC: Area under the curve
PPV: Positive predictive value
NPV: Negative predictive value
